# Predicting Signatures of “Synthetic Associations” and “Natural Associations” from Empirical Patterns of Human Genetic Variation

**DOI:** 10.1371/journal.pcbi.1002600

**Published:** 2012-07-05

**Authors:** Diana Chang, Alon Keinan

**Affiliations:** 1 Department of Biological Statistics and Computational Biology, Cornell University, Ithaca, New York, United States of America; 2 Program in Computational Biology and Medicine, Cornell University, Ithaca, New York, United States of America; Columbia University, United States of America

## Abstract

Genome-wide association studies (GWAS) have in recent years discovered thousands of associated markers for hundreds of phenotypes. However, associated loci often only explain a relatively small fraction of heritability and the link between association and causality has yet to be uncovered for most loci. Rare causal variants have been suggested as one scenario that may partially explain these shortcomings. Specifically, Dickson et al. recently reported simulations of rare causal variants that lead to association signals of common, tag single nucleotide polymorphisms, dubbed “synthetic associations”. However, an open question is what practical implications synthetic associations have for GWAS. Here, we explore the signatures exhibited by such “synthetic associations” and their implications based on patterns of genetic variation observed in human populations, thus accounting for human evolutionary history –a force disregarded in previous simulation studies. This is made possible by human population genetic data from HapMap 3 consisting of both resequencing and array-based genotyping data for the same set of individuals from multiple populations. We report that synthetic associations tend to be further away from the underlying risk alleles compared to “natural associations” (i.e. associations due to underlying common causal variants), but to a much lesser extent than previously predicted, with both the age and the effect size of the risk allele playing a part in this phenomenon. We find that while a synthetic association has a lower probability of capturing causal variants within its linkage disequilibrium block, sequencing around the associated variant need not extend substantially to have a high probability of capturing at least one causal variant. We also show that the minor allele frequency of synthetic associations is lower than of natural associations for most, but not all, loci that we explored. Finally, we find the variance in associated allele frequency to be a potential indicator of synthetic associations.

## Author Summary

Genome-wide association studies (GWAS), based on the hypothesis that common genetic variation underlies complex diseases, have found many sites in the genome associated with complex diseases. However, both the fraction of variation explained by these sites and the number of studies identifying causal variants remain low. While there are many possible explanations for these issues, we focus on one theory that suggests rare variants also play a significant role in complex diseases. We investigated the effect of rare causal variants as compared to common causal variants in simulated data with patterns of variation observed in actual human genetic data. As suggested by previous studies, we found that rare causal variants show different signatures in GWAS results. We explore in this study the implications of these differences in influencing the search for causal variants underlying the signal of association.

## Introduction

Recent years have seen a plethora of genome-wide association studies (GWAS) finding thousands of common markers that are associated with hundreds of diseases and other traits. GWAS were initially founded on the Common Disease-Common Variant hypothesis [1]–[3], which predicted that common complex diseases are most likely caused by a few common variants. As a consequence, the design of most GWAS consisted of genotyping common tag single nucleotide polymorphisms (SNPs) and comparing their allele frequencies between cases and controls. Some limitations of this design have been the topic of much recent discussion, with the gap between association and causality and the relatively small portion of heritable variation explained by associated markers drawing the most concern [4]–[7]. Several hypotheses aiming to explain the missing heritability have been proposed, including the roles of structural variants, gene-gene interactions, gene-environment interactions, epigenetics, and complex inheritance [4]–[7]. In addition, rare variants of relatively high penetrance contributing to disease risk [8], [9] has also been suggested as a source of missing heritability since rare variants have not been directly observed in most GWAS, and they might be differently tagged by common markers [10]–[12].

Given this renewed interest in such variants, an investigation into their effect on GWAS association signals is warranted. A recent simulation-based study showed that rare causal variants can often create “synthetic associations,” namely significant associations of common markers induced by the combined effect of one or more rare causal variants [13]. Dickson et al. further showed that a synthetically associated common marker could be substantially further away than expected had the underlying causal variant been common, and that synthetic associations are expected to be on average of lower minor allele frequency (MAF) than associations due to underlying common causal variants [13]. These predictions may partially explain why resequencing fine-mapping efforts, which are based on patterns of linkage disequilibrium (LD) of common variants, have often been unsuccessful in uncovering causal variants [10], [13], [14]. As the development of new methods and study designs for associating rare causal variants is underway [12], [15]–[24], the predictions of Dickson et al. are influencing the choice of study design, as well as the interpretation of traditional, genotyping-based GWAS (e.g. [25], [26]).

A few instances of rare causal variants have already been well established [27]–[29], including potentially causal rare variants in NOD2 that contribute to Crohn's disease risk [30]–[33]. In this example, since an associated common marker in the same gene is in LD with at least two of the rare variants, it is possible that they contribute to the marker's association signal [30], thus inducing a synthetic association. As only a few examples of rare causal variants contributing to complex disease are well established, the jury is still out on their prevalence and on how often they lead to synthetic associations, with several recent studies arguing that the phenomenon is not necessarily widespread [34]–[36]. In light of this uncertainty, a detailed investigation of the signatures of synthetic associations and their implications is crucial for interpreting the results of genotyping-based GWAS and for considering the alternative of association studies based on whole-genome or whole-exome sequencing.

Two of the key questions with regards to “synthetic associations” are (1) what are the implications for the resequencing distance for fine-mapping of significant associations? and (2) how different is the MAF of synthetic associations from that of “natural associations” (i.e. associations where the underlying causal variants are common)? While these questions have been addressed in studies of simulated data [13], [36], those simulations did not account for the nature of disease loci and risk variants, nor did they account for the specific nature of human genetic variation. In the former, it has been shown that the effect size and frequency of the disease variants can alter the power of the test [37]. While, in the latter, the mark left by human evolutionary history on patterns of genetic variation can greatly influence the nature of significant association signals, which we address in the present study. For example, when considering samples from European populations, which have been the populations of choice of most GWAS, it is crucial to account for their recent explosive population growth that has led to an inflation in the proportion of rare variants and to an altered haplotype and LD structure [38]–[41], as well as to account for the well-established effects of the earlier Out-of-Africa event on these genetic patterns [42]–[48].

Here, we focus on the question of how empirical LD patterns can affect signals of “synthetic association” by investigating them in real human population genetic data. Through this, we aim to derive a better understanding of synthetic associations and their practical implications. Using empirical resequencing data, we randomly assume certain variants as increasing disease risk, determine cases and controls accordingly, and conduct an association study using genotyping data of the same individuals from arrays that have been employed in most GWAS. To illuminate and quantify signatures that are specific to “synthetic associations”, we repeat the process for rare and common causal variants and contrast the characteristics of synthetic associations with those of natural associations.

We aim to elucidate how far associations are from the underlying causal variants, how their frequencies are distributed and, more importantly, how these different signatures should alter the design of fine-mapping studies. To examine possible heterogeneity in these signatures across the genome and across populations with different evolutionary histories, we repeated the analysis for several resequencing loci on different chromosomes and for two populations, one West African and one North European. The novelty of this study is in elucidating implications of synthetic associations and how they may affect fine-mapping strategies with patterns of LD as observed in human populations.

## Results

To empirically investigate the signatures of “synthetic associations”, we needed to examine scenarios in human genetic data where the presumed disease risk variants—rare or common—are known. Thus, we considered “disease loci” in the ENCODE regions that were sequenced as part of HapMap 3 [49]. The advantages of using these resequencing data are overcoming ascertainment biases that plague genotyping arrays [45], [50]–[52] and observing variants of much lower allele frequency. Equipped with resequencing data for over 110 individuals in each population, we studied variants that appeared at least twice in 220 chromosomes. We randomly assigned variants within each disease locus as being causal and considered individuals carrying any one of these variants to have elevated disease risk. We then probabilistically assigned individuals to be either cases or controls based on their assigned risk. To mimic the case of many rare variants of large effect size underlying synthetic associations, and to contrast it with that of a few common variants of moderately low effect sizes underlying natural associations, we investigated three scenarios: (i) 2 common causal variants with a genotypic relative risk (GRR) of 1.5, (ii) 5 and (iii) 9 rare causal variants with a genotypic relative risk of 3. We verified that our results are not an artifact of the number of causal variants, as illustrated in the following, by comparing with a less realistic scenario of 5 common causal variants. We also considered a random assignment of cases and controls, which provides a null distribution in the absence of any risk alleles.

After obtaining a set of cases and a set of controls, we performed an association study using the genotyping array data for the same individuals from HapMap 3 [49], without considering any of the resequencing data in which disease loci have been emulated (Materials and Methods). This mimics the conditions and variant-type of actual genotyping-based GWAS, which typically utilize array data of mainly common markers, most often using the same or similar arrays to those we have used for our analyses (a combination of Affymetrix Human SNP array 6.0 and Illumina Human1M). We report results for association testing of all genotyped markers located within 3 cM of the resequenced disease locus, after verifying that the vast majority of significant associations are within those bounds (Materials and Methods). Similar to the requirement of genome-wide significance in a GWAS, we required significance following multiple-hypothesis correction for the entire region tested, such that our results can be extrapolated to genome-wide studies. We repeated the association testing for 5 different disease loci (Table 1) and for 50 sets of random assignments of causal variants in each locus. For each of these sets, we repeated the association testing in 10 replicates, varying between them only the stochastic assignment of cases and controls, for a total of 500 association tests in each locus for each of the three scenarios of causal variants. We also considered separately both a European (CEU) and a West African (YRI) population. Because of the relatively small sample size of ∼110 individuals, we simulated a larger sample using HAPGEN [53], which maintains the genetic variation observed in the original data, including patterns of LD and MAF (Materials and Methods).

**Table 1:** List of ENCODE regions used as disease loci [45].

Locus #	ENCODE name	Chromosome	Location (bp)	# Common variants* (YRI/CEU)	# Rare variants* (YRI/CEU)
1	ENr221	5	56071684	57/36	59/20
			−56170943		
2	ENm010	7	27124056	58/40	117/57
			−27223436		
3	ENr321	8	119082399	72/20	108/45
			−119182123		
4	ENr123	12	38827200	43/62	72/50
			−38925373		
5	ENr213	18	23920590	60/54	108/41
			−24019175		

*: Variants with MAF of either between 0.1–0.3 or between 0.005–0.04 after resampling of haplotypes using HAPGEN.

**Figure 1:**
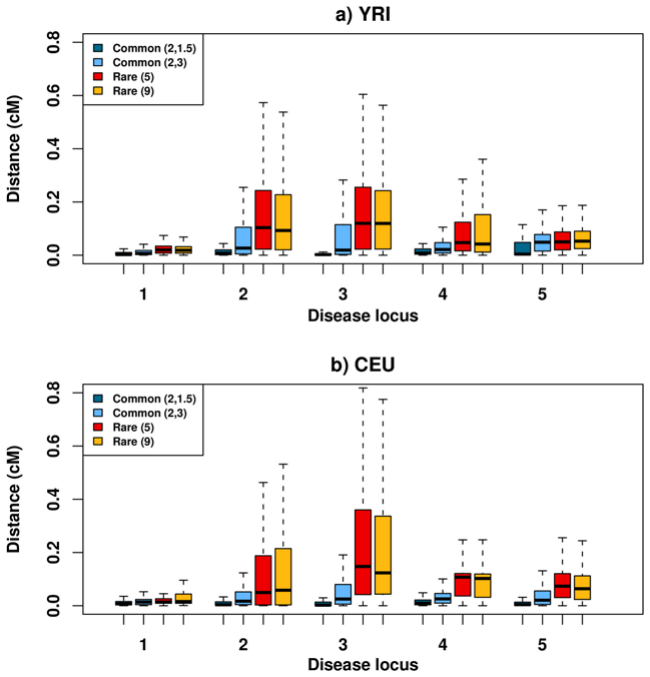
Distance of synthetic and natural associations from the causal variant it is in greatest LD with. Box plot of the distance between any associated SNP and causal variant it is in highest LD with, measured in *r^2^*, for (**a**) YRI and (**b**) CEU in four scenarios: 2 common causal variants with a GRR of 1.5 (dark blue), 2 common causal variants with an unrealistic GRR of 3 (light blue), 5 and 9 rare causal variants with a GRR of 3 (red and gold respectively). Distances vary greatly between the different disease loci (x-axis) as well as between populations, but in all regions the median (line within each box) is larger for rare causal variants than for common causal variants of lower effect size. Increasing the effect size can result in higher association distance as is observed most notably in region #5.

All scenarios show significant associations much more often than the false discovery rate of 5% (Table S1). To determine whether “synthetic associations” due to underlying rare variants tend to be further away than “natural associations” due to underlying common variants, we considered for each association test the distance between any association and the causal variant with which it is in strongest LD (Materials and Methods). We found that the median distance, over the many hundreds of associations found across the 500 tests, is variable across the five loci and—to some extent—between the two populations (Figure 1). Synthetic associations tend to be much further than natural associations, as previously predicted [13], though for one region (disease locus #1) both synthetic and natural associations are in close proximity to the causal variants (Figure 1). Alternatively, when considering the distance between an association and the closest causal variant (rather than the one in strongest LD), the distance of synthetic associations is reduced, yet generally remains greater than that of natural associations (Figure S1). Taken together, these results lead us to ask what factors contribute to this increased distance, and, more importantly, to what extent this increased distance should impact the choice of fine-mapping strategies.

We explored several plausible explanations for this increased distance. Firstly, we ensured that the increased distance of rare causal variants is not due to more variants in those scenarios (5 and 9) than in the scenario of common causal variants (2) by repeating our analysis for cases with 5 common causal variants. We observed no increase in association distance of resultant natural associations (Figure S2), revealing that the increased distance is not due to the increased number of causal variants. Secondly, we investigated the hypothesis that increased marker effect size can cause greater association distances since association power is proportional to effect size times the correlation between the causal variant and the marker [37]. We investigated this hypothesis by increasing the effect size of common causal variants to equal that in the scenario of rare causal variants, though such an effect size might be considered unrealistic for common variants. The median association distance of the resulting natural associations indeed increases for all regions and populations, but is still considerably lower than synthetic associations in most cases (Figure 1).

**Figure 2:**
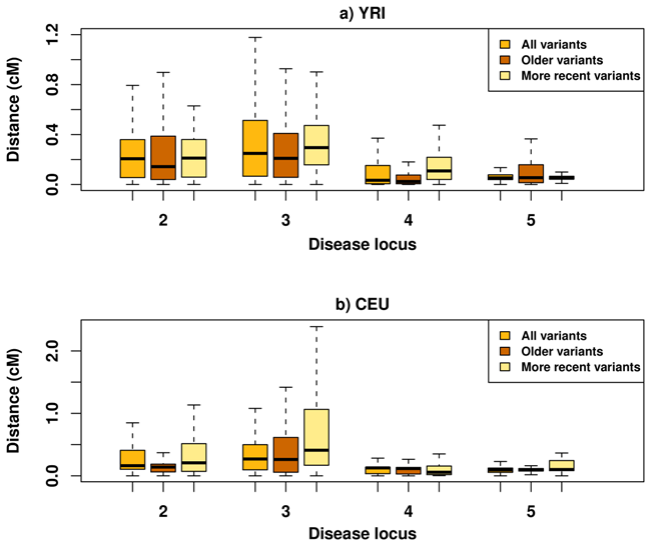
Distance of causal variant from “synthetic associations” partitioned by the age of the mutation. Box plot similar to Figure 1, while separating rare variants in CEU and YRI into a *more recent* and an *older* class (Materials and Methods). Variants due to more recent mutations result in much increased distance between the associated SNP and the causal variant with highest LD in 3 regions in YRI and 2 regions in CEU. Results are presented for only 4 of the disease loci due to lack of relevant data in locus #1. Note that the risk allele frequency range for rare variants is narrower compared to Figure 2 (Materials and Methods) and that the y-axis scale is different between the two populations.

We next tested whether the age of the mutation played a role in increasing association distances for synthetic associations. As rare variants are, on average, resultant of more recent mutations compared to common variants, recombination would have had less time to operate, thus resulting in diminished decay of LD and haplotype structure around rare variants [39]. To test whether the age of the mutation plays a part in explaining our results, we partitioned rare causal variants into two age groups: i) variants due to relatively *more recent* mutations and ii) variants due to relatively *older* mutations. Variants with minor alleles present in only a single population fell into the former category, while those with minor alleles present in more than one population fell into the latter (Materials and Methods). We observed a larger distance between an associated marker and the causal variant with which it is in highest LD for *more recent* mutations than for *older* mutations (Figure 2). Out of the 4 disease loci for which enough data was available to perform this analysis, 3 in YRI and 2 in CEU exhibit a median distance from *older* rare causal variants that is at least 41% less than the median distance from *more recent* causal variants. Combined, these results suggest that the increased distance of synthetic associations compared to natural associations is partially due to the young age of the mutations that give rise to rare risk alleles, as well as due to the higher effect size that is claimed to be implicated for rare risk alleles.

**Figure 3:**
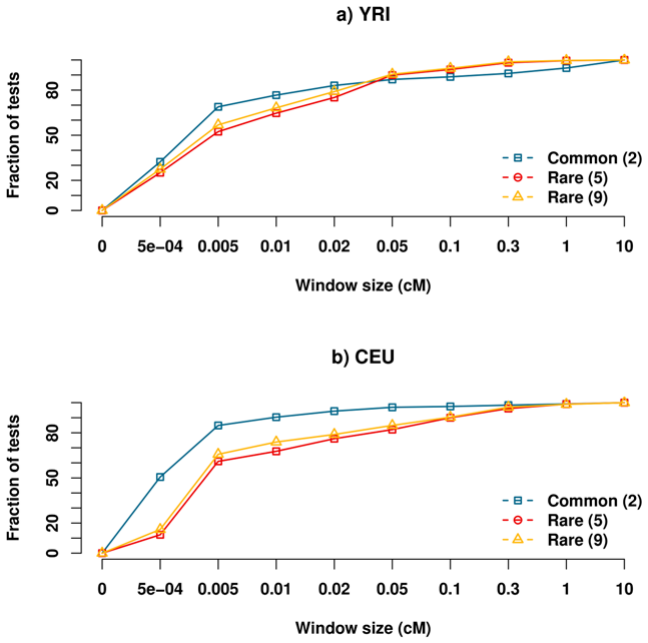
Resequence window size necessary to capture at least one causal variant. The figure presents for a given window size, the fraction of tests combined over all regions with significant associations where at least one association is within the given distance from the causal variant it is in highest LD with. The colors correspond to the same scenarios as in Figure 1. Resequencing need not extend much further than in the common causal variant case, as a window of size of 0.1 cM has at least one association tagging a rare causal variant in >90% of the tests between both populations and all regions.

The main concern regarding synthetic associations is how their signatures alter the search for the actual causal variant(s). Specifically, how far should one sequence around an association in order to capture causal variants? We addressed this question using two approaches. We first computed for each scenario of causal variants the fraction of tests (out of all tests with any significant association) that had at least one associated marker within any given distance of the causal variant with which it is in highest LD. We found that for common causal variants, a shorter resequencing distance of 0.01 cM is enough to capture a causal variant in 90% of the tests in CEU and 77% for YRI (Figure 3). For rare causal variants, combined over all disease loci, at least 90% of tests discovered an association within 0.1 cM of a causal variant (Figure 3). Secondly, we investigated a scenario in which fine-mapping consists of sequencing the LD block of associations as observed in the data. Hence, we estimated the probability that an associated marker is in the same LD block as any of the causal variants, with the definition of LD blocks being based only on markers from the genotyping arrays, which are relatively common (Materials and Method). On average, the LD blocks spanned 0.007 cM for CEU and 0.005 cM for YRI, after the addition of a flanking region of 0.0005 cM. We found that in CEU, 94% of associated markers had a common causal variant in the same LD block, while the same was true for only 78% of associated markers in the rare causal variant case. A similar trend was observed for YRI, albeit less marked, where 79% of natural associations captured a causal variant, but only 73% of synthetic associations captured a causal variant.

**Figure 4:**
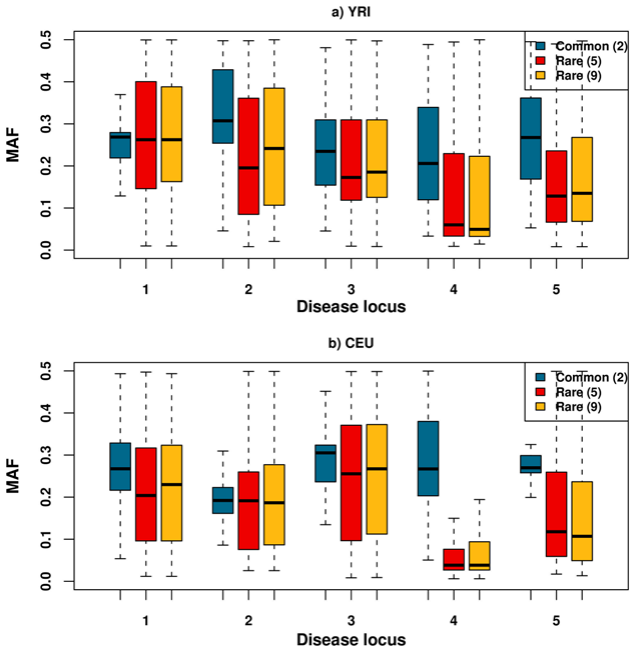
Minor allele frequency (MAF) of associated variants. Box plot of the minor allele frequency for all associated variants in the different scenarios. Although synthetic associations have median MAF lower than that of natural associations, the range of MAF for synthetic associations varies across the different loci and populations. The median MAF is similar between the natural and synthetic associations for a few loci (disease locus #2 in CEU and #1 in YRI).

Finally, we explored the minor allele frequency (MAF) of associated markers and found that natural associations are of higher frequency on average than synthetic associations (Figure 4). Summing over all disease loci and populations, <1% of natural associations had MAF below 0.1, while this proportion increased to 15–28% for synthetic associations. Dissecting the signal further by region and population, we found that while some regions display less than 2.4% difference between the median MAF of natural associations and synthetic associations (disease locus #1 in YRI, #2 in CEU), others display an almost 200% difference (#4 in CEU). Synthetic associations also display a larger standard deviation in associated MAF across different associations in different sets and replicates as compared to natural associations, with all but one region displaying a difference ranging from 17%–70% (Table 2).

**Table 2:** Standard deviation of minor allele frequency for associated variants.

Locus #	Common (2)	Rare (5)	Rare (9)
**YRI**			
1	0.086	0.134	0.131
2	0.117	0.154	0.150
3	0.114	0.131	0.124
4	0.124	0.151	0.145
5	0.113	0.121	0.126
**CEU**			
1	0.084	0.113	0.116
2	0.056	0.118	0.121
3	0.064	0.152	0.143
4	0.121	0.121	0.126
5	0.073	0.133	0.136

## Discussion

With the use of HapMap 3 resequencing and genotyping data from five different genomic regions and two populations [49], we considered several scenarios of disease risk loci, and performed association tests to investigate the signatures of synthetic associations and how they alter one's approach for studying them. We found that the median distance of synthetic associations, while greater than that of natural associations, still never exceeds 0.15 cM (∼150 kb) for any of the 10 locus-by-population settings. Even if we instead consider the worst-case scenario of the largest distance between any association and any causal variant, its median still never exceeds 0.41 cM (∼410 kb). These results are in clear contrast to the results of a previous simulation-based study that showed the median of the largest distance to be 5 cM (5 Mb) [13]. The difference between the two studies may be attributed to differences in the frequencies of rare causal variants. We considered rare alleles of frequency in the range 0.005–0.04 (average across all variants of 0.019), while Dickson et al. simulated allele frequencies in the range 0.005–0.02 [13] (average of 0.0125 assuming uniform sampling). However, when we restricted to a narrower range of frequencies up to 0.02 (average of 0.012), we still observed no locus for which the median distance of synthetic association exceeds 0.5 cM (‘All variants’ in Figure 2). It is unlikely that any remaining slight difference in risk allele frequency would result in over an order of magnitude difference in association distance.

A more substantial difference between the two studies lies in the data analyzed. Dickson et al. conducted simulations of constant effective population size, uniform recombination rate, and neutral loci, with association testing based on a simulated “genotyping array” that follows a uniform ascertainment bias [13]. Here, we have analyzed data with empirically observed LD patterns, and have based association testing on data from real genotyping arrays as designed for GWAS. Put together, while theory posits that a median distance of synthetic associations of 5 cM is possible, characteristics of empirical data suggests that such cases will not be common, and that even under the worst-case scenario the vast majority of synthetic associations are at least an order of magnitude closer.

By considering which of the rare polymorphisms are population-specific, and hence likely to be more recent, we illustrated that the increase in association distance is partially due to the age of the mutation. This is likely a result of recombination having had less time to break down the haplotype surrounding more recent mutations. We also considered common causal variants with a higher effect size and showed that association distance is increased. As rare causal variants contributing to an association signal are claimed to have higher effect sizes than common causal variants, the increased distance for synthetic associations can thus partially be due to the larger effect size. Additionally, the contribution of multiple rare causal variants to a single signal of association may also increase association distance –a source we have yet to fully explore in detail.

To assess the impact of this increased association distance, we explored the probability that an association test had at least one association where the causal variant with which it was in highest LD lay within a given distance from the association. We found that for rare causal variants a window size of 0.1 cM was sufficient to capture at least one causal variant in such a manner in at least 90% of the tests for all regions and populations (Figure 3). Alternatively, by following an LD block based approach for fine-mapping, 73–79% of synthetic associations capture at least one of the rare causal variants within the same LD block. This suggests that traditional LD block-based fine-mapping offers a pretty high probability of discovering some of the causal variants, though there could still be added benefit from sequencing a larger region. Preliminary analysis suggests that it is difficult to predict the optimal region to resequence given a specific disease locus, as no single factor, such as pair-wise LD decay, can sufficiently predict this distance (data not shown). Further work is thus necessary in order to determine which factors that influence synthetic associations, such as the age of mutation, causal variant effect size, haplotype structure and the stochastic coupling of multiple rare variants on the background of a common marker, play a role in an observed association signal.

In a further analysis, we found that the causal variants being rare entails that the associated markers will themselves be of lower frequency (Figure 4), a result consistent with previous simulation studies [13], [36]. When narrowing the number of associations to only the most significant, we found that this further reduced the allele frequency of synthetic associations (Figure S3). In addition, we found that the frequency of synthetic associations often had a larger standard deviation than natural associations (Table 2). These results have two implications. Firstly, it suggests that synthetic associations as compared to natural associations are likely to have underestimated effect sizes of the causal variant due to reduced associated allele frequencies [54] (especially when analyzing the most significant association) and from incomplete LD with the causal variant. Secondly, this suggests that the standard deviation of the associated minor allele frequency can offer a way to flag for underlying rare causal variants that induce potential synthetic associations; given a larger standard deviation of associated frequencies, it would be advised to follow a wider fine-mapping study design.

Due to the >1000-fold human population growth in the last hundreds of generations, the amount of rare variation is much greater than expected [38]–[41]. This explosive addition of rare variation entails an LD structure that is yet to be quantified, but certainly disparate than the extensively studied LD structure of common variants. In addition, the earlier founder events as modern humans migrated out of Africa and settled across the globe have been shown to greatly alter patterns of genetic variation [42]–[45], [55]. For this reason we studied both a West African population and a population of European ancestry, with differences in our results between the two reinforcing the importance of taking demographic history into consideration by studying empirical data. The effect of evolutionary history on signatures of synthetic and natural associations is further supported by the highly variable behavior across genomic regions of all the signatures we observed.

In conclusion, this study delivered a characterization of several signatures of synthetic associations and assessed their impact on the search for the causal variant(s) underlying the signal. While our study does not take part in the debate on how frequently synthetic associations occur, it is relevant in any situation in which they do. We illustrated that because synthetic associations are likely to be more distant from causal variants, fine-mapping studies should look further than when searching for common causal variants, but to a much lesser extent than previously suggested. We also propose the larger standard deviation of associated allele frequencies as a way to detect potential rare causal variants at play. Additional analysis is warranted though, to elucidate the quantitative relationship between genetic architecture, demographic history, allele frequency and association signals. Finally, although the debate remains open as to the contribution of rare risk alleles to human complex diseases and to the ensuing abundance of synthetic associations [34]–[36], [56], our results offer new guiding principles for determining a length of a region to fine-map, and for considering the alternative of an association study based on whole-genome or whole-exome sequencing.

## Materials and Methods

### Data

We obtained from HapMap 3 [49] genotyping array data for YRI (Yoruba in Ibadan, Nigeria) and CEU (individuals in Utah with Northern and Western European ancestry from the Centre d'Etude du Polymorphisme Humain collection) and resequencing data of five ENCODE regions, each 100 kb in length (Table 1), for 115 YRI and 111 CEU individuals. We also obtained resequencing data for 60 TSI (Toscani in Italia) samples and 60 LWK (Luhya in Webuye, Kenya), which we used for the *variant age analysis* (below). We considered each resequencing region as a disease locus from which to select causal variants. Using resequencing data facilitates higher concentration of rare variants and is free of the ascertainment biases associated with genotyping arrays [45], [50]–[52].

### Simulated Data

Due to the low sample size, we employed HAPGEN [53] to simulate 10,000 individuals for each population –a strategy previously employed to investigate the estimation of relative risks [54]. HAPGEN simulates additional haplotypes by treating each new haplotype as a mosaic of already present haplotypes. We refer readers to [53] for additional details on HAPGEN.

We first phased and imputed missing data with BEAGLE v3.3 [57]. We then simulated additional data for each resequencing region and the 3 cM-flanking window for each region using HAPGEN with a recombination map from the March 2006 human reference sequence (NCBI Build 36, hg18) and a null mutation rate as input parameters. We ensured that the LD patterns of the original data (for rare and common variants) were maintained (Figure S4). We also ensured that allele frequencies in the simulated data do not change drastically from the original data as no variants were observed that were initially of very low frequency and attained a much higher frequency and vice versa in the simulated dataset (Figure S5, S6).

Association tests were performed using the simulated data from the HapMap 3 genotyping array data, excluding any causal variants that happen to be in the genotyping array data. We report results for an association study for SNPs located in the disease locus and in flanking regions of 3 cM on each side (from which no causal variants are chosen), as almost no associations were observed to fall beyond that distance (data not shown). In our study, rare causal variants have risk allele frequencies in the simulated data between 0.005 and 0.04 (we note that a portion of this range is defined as “low frequency”, rather than rare, by some studies), and common causal variants have risk allele frequencies in the simulated data between 0.1 and 0.3. In testing for association, we considered all SNPs of all allele frequencies from the genotyping data. All coordinates and genetic distances in this paper are according to the March 2006 human reference sequence (NCBI Build 36, hg18).

### Disease Model and Association Study Design

We considered each individual as a case or a control with a probability proportional to the individual's assigned risk, which is elevated if the individual has one or more risk alleles. We set the baseline risk as 0.15 and the genotypic relative risk to 1.5 for the scenario of common causal variants. We also explored an unrealistic genotypic relative risk of 3 for common causal variants to investigate the influence of effect size on association distance. For rare causal variants, we assigned a higher genotypic relative risk of 3. While the use of a fixed GRR for variants of differing allele frequencies results in differing portions of variance explained by each variant, it is a more realistic disease model. By fixing variance explained, rarer variants would tend to have higher, and perhaps somewhat unrealistic, GRRs. Because we have fixed GRR and allowed the proportion of variance explained to vary, an association test will have more power in detecting variants of higher allele frequency given a fixed GRR.

For the common causal variants scenario, we randomly assigned 2 SNPs from the resequencing data as causal, while we assigned either 5 or 9 for the rare causal variants scenario. To ensure that the number of causal variants did not affect our results, we also studied a scenario with 5 common causal variants in loci where this was feasible. For each scenario of a certain type and number of causal variants, 50 sets of causal variants were randomly selected, with replacement between groups. Each of these 50 sets allows for a possibly different risk for each individual. For each of these 50 sets, we repeated 10 replicates of randomly assigning cases and controls according to the same individual assigned risk.

In each of the 500 association tests (50 different variant groups and their 10 phenotypic replicates), we randomly chose 1000 cases and 1000 controls according to the individual's assigned risk. This ensures that the same number of cases and controls were shared across all analyses, thereby having comparable statistical power. For each scenario of type and number of causal variants, we pooled together the results from these 500 tests for the statistics and figures presented in this study. Similarly, we generated 500 tests for each disease locus with randomly assigned case/control status to serve as a control.

All association tests were done with PLINK's logistic regression function [58]. Significance thresholds were determined with a region-wide Bonferroni correction. For the control scenario of random assignment of cases and controls, 2.12% of the association tests showed a significant association as compared with the expectation of 5%.

### Distance Analysis

We determined genetic distances based on the Oxford genetic map based on HapMap2 data [50], [59]. For SNPs missing from HapMap2, we estimated the position as the linear interpolation of the genetic positions of the two closest SNPs. The association distances were determined by computing the genetic distance between an associated SNP and the causal variant with which it was in highest LD, measured in *r^2^*. Pairwise *r^2^* values were calculated in pLINK [58].

### Age of Mutation Analysis

To partition rare variants based on the age of the mutation, we first narrowed the range of the risk allele frequency in the simulated data to 0.005 and 0.02 in order to ensure a roughly equal partition into the two age groups. We discarded disease locus #1 from this analysis because it had too few rare variants to allow their portioning into two groups (Table 1). Rare variants in the 111 CEU individuals were defined to be relatively *more recent* if only the major allele was observed in the resequencing of 115 YRI individuals and 60 TSI individuals in the original data; the variant was defined as relatively *older* otherwise. We repeated the above analyses for each of these groups separately, such that in each association testing either all causal variants are *older* or all are *more recent*. We repeated the same analysis in YRI with CEU and 60 LWK as out groups. We duly note that polymorphisms absent from the limited number of samples may not be monomorphic in the population as a whole, hence not all mutations leading to relatively *older* variants precede those leading to variants in the relatively *more recent* class. Yet, this represents only a small fraction of variants and variants in the relatively *older* class are expected to be older on average than those belonging to the *more recent* class. It is also important to note that false positive variant calls are added to the *more recent* group despite the erroneous call. This scenario is highly unlikely in our analyses due to the stringent quality control measures taken in HapMap 3 [45] and the exclusion of singletons in our study. For each of these two scenarios of causal variants, we similarly chose 50 sets of causal variant groups with 10 phenotypic replicates each and obtained maximal distances as above. For comparison, we repeated the analysis for random rare causal variants in the narrowed range of frequency of 0.005–0.02 used here, irrespective of mutation age.

### Resequencing Distance Analysis

For each association test we explored whether a causal variant with which an association is in highest LD (measured in *r^2^*) is within a given genetic distance from the association. For each simulated scenario and resequencing window size ranging from 0 cM to 10 cM, we calculated the proportion of tests that have at least one such association.

For the second analysis, we observed over all significant associations if any causal variant was in the same LD block as an association. LD blocks were estimated in pLINK with the genotyping data [58] and 0.0005 cM was added to the start and end coordinates in order to compensate for the uncertainty in these estimates.

## Supporting Information

Figure S1 **Distance between association and closest causal variant.** The figure mirrors Figure 1, but plots instead the distance between an association and the closest causal variant. The distance of synthetic associations is reduced, yet generally remains greater than that of natural associations.
(TIFF)

Figure S2 **Distance of common causal variant is not sensitive to the number of causal variants.** The figure mirrors Figure 1, but to the inclusion of results for 5 common causal variants (“Common (5)”) in loci where this was feasible (all for CEU). All other results are reproduced from Figure 1. The difference in distance between common and rare causal variants remains even with 5 common causal variants.
(TIFF)

Figure S3 **Minor allele frequency of most significant association.** The figure mirrors Figure 4, but displays the minor allele frequency of only the most significant association across each test. The median frequency of the most significant association is reduced for synthetic associations.
(TIFF)

Figure S4 **Empirical LD patterns are preserved in HAPGEN simulations.** Plotted above is data for region 1 in CEU. For each 0.01 cM bin, the figure presents the mean pair-wise LD (measured in r^2^) between variants from the resequencing and genotyping data for a) common markers (minor allele frequency >0.04) or b) common and rare markers (minor allele frequency <0.04). We observe that HapMap 3 LD patterns (blue) are largely preserved in HAPGEN simulations (green). Missing points reflect lack of data for certain distance bins.
(TIFF)

Figure S5 **Minor allele frequency in HapMap3 compared to minor allele frequency in HAPGEN simulations.** Plotted are minor allele frequencies in HapMap 3 (x-axis) compared to minor allele frequencies in HAPGEN simulations (y-axis) for a) YRI and b) CEU. Each row represents a separate region. No drastic departures from the original minor allele frequencies are observed in the simulated data.
(TIFF)

Figure S6 **Minor allele frequency in HapMap3 compared to minor allele frequency in HAPGEN simulations for frequencies below 0.08.** Same plot as in Figure S5 showing only variants with frequencies below 0.08. As in Figure S5, no drastic departures from the original minor allele frequencies are observed in the simulated data.
(TIFF)

Table S1 **Percentage of tests with significant associations.**
(DOC)
