# Supplementary figures and images for: Predicting Signatures of “Synthetic Associations” and “Natural Associations” from Empirical Patterns of Human Genetic Variation
Source: PLoS Comput Biol. 2012 Jul 5;8(7):e1002600. doi: 10.1371/journal.pcbi.1002600 (PMC3390358; doi:10.1371/journal.pcbi.1002600)

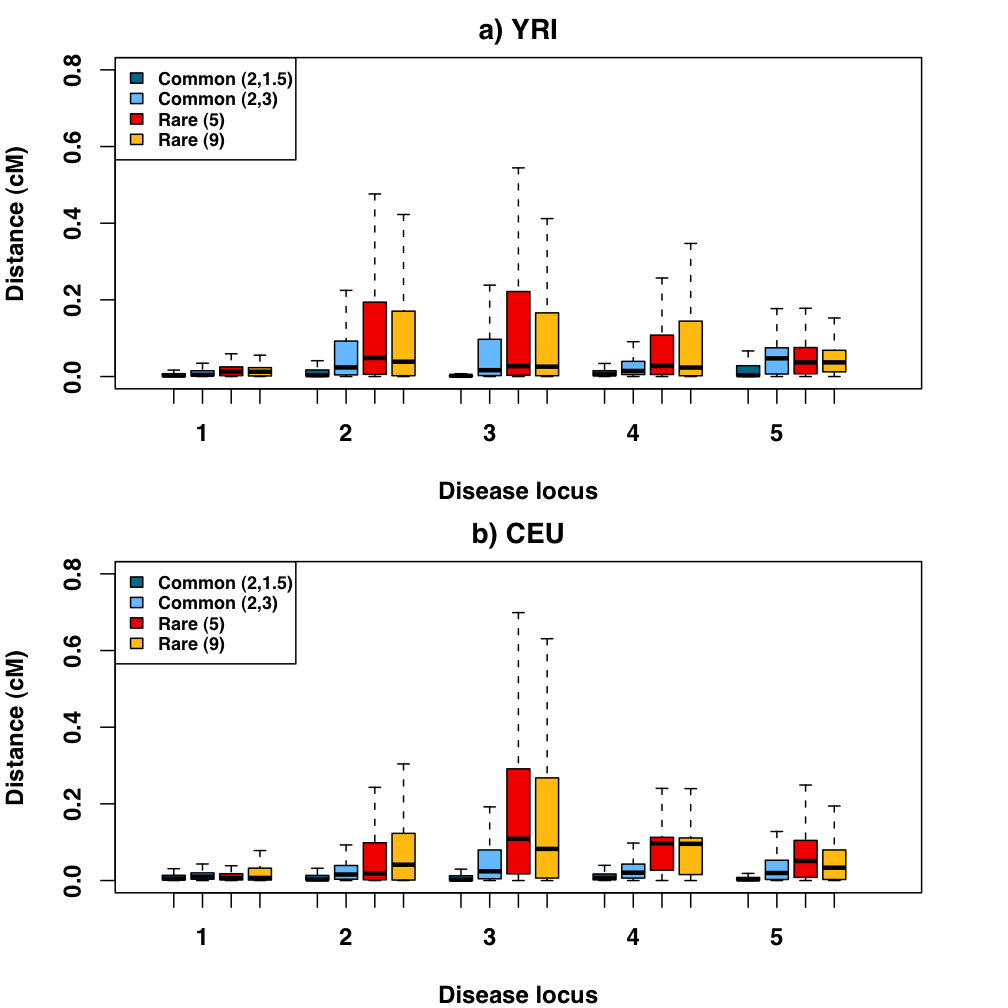

Supplement: Figure S1 — Distance between association and closest causal variant. The figure mirrors Figure 1, but plots instead the distance between an association and the closest causal variant. The distance of synthetic associations is reduced, yet generally remains greater than that of natural associations. (TIFF) [file pcbi.1002600.s001.tif]

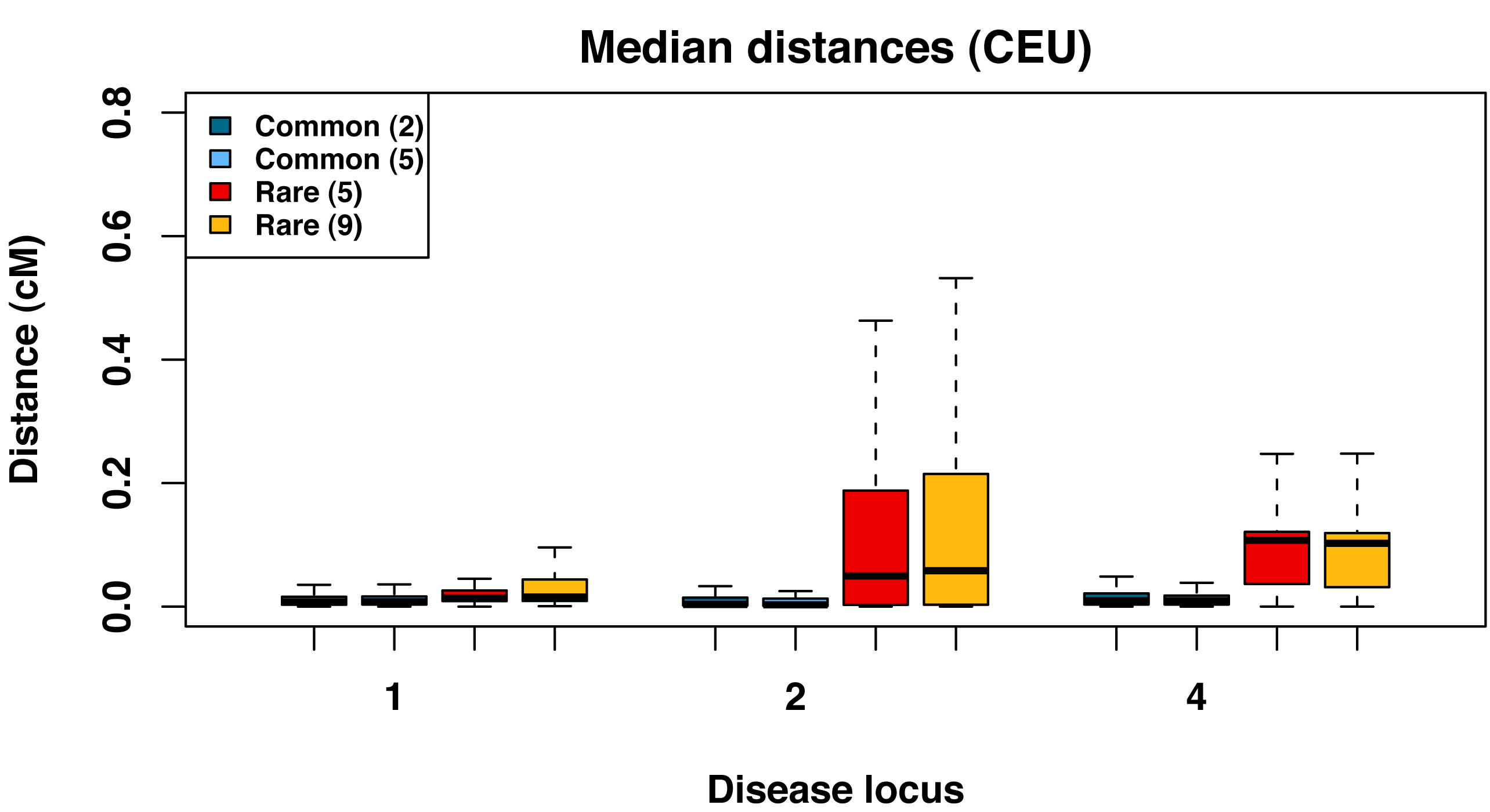

Supplement: Figure S2 — Distance of common causal variant is not sensitive to the number of causal variants. The figure mirrors Figure 1, but to the inclusion of results for 5 common causal variants (“Common (5)”) in loci where this was feasible (all for CEU). All other results are reproduced from Figure 1. The difference in distance between common and rare causal variants remains even with 5 common causal variants. (TIFF) [file pcbi.1002600.s002.tif]

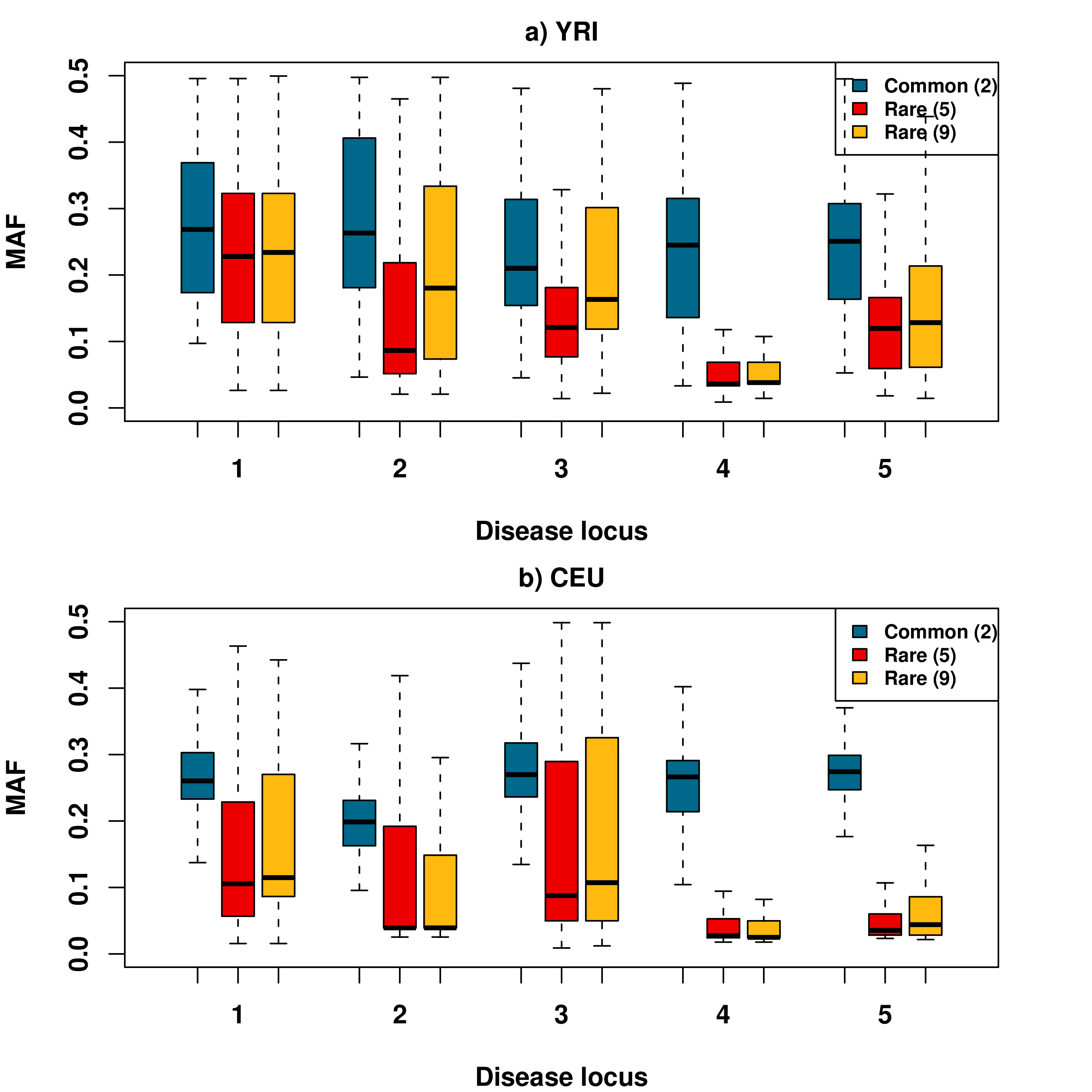

Supplement: Figure S3 — Minor allele frequency of most significant association. The figure mirrors Figure 4, but displays the minor allele frequency of only the most significant association across each test. The median frequency of the most significant association is reduced for synthetic associations. (TIFF) [file pcbi.1002600.s003.tif]

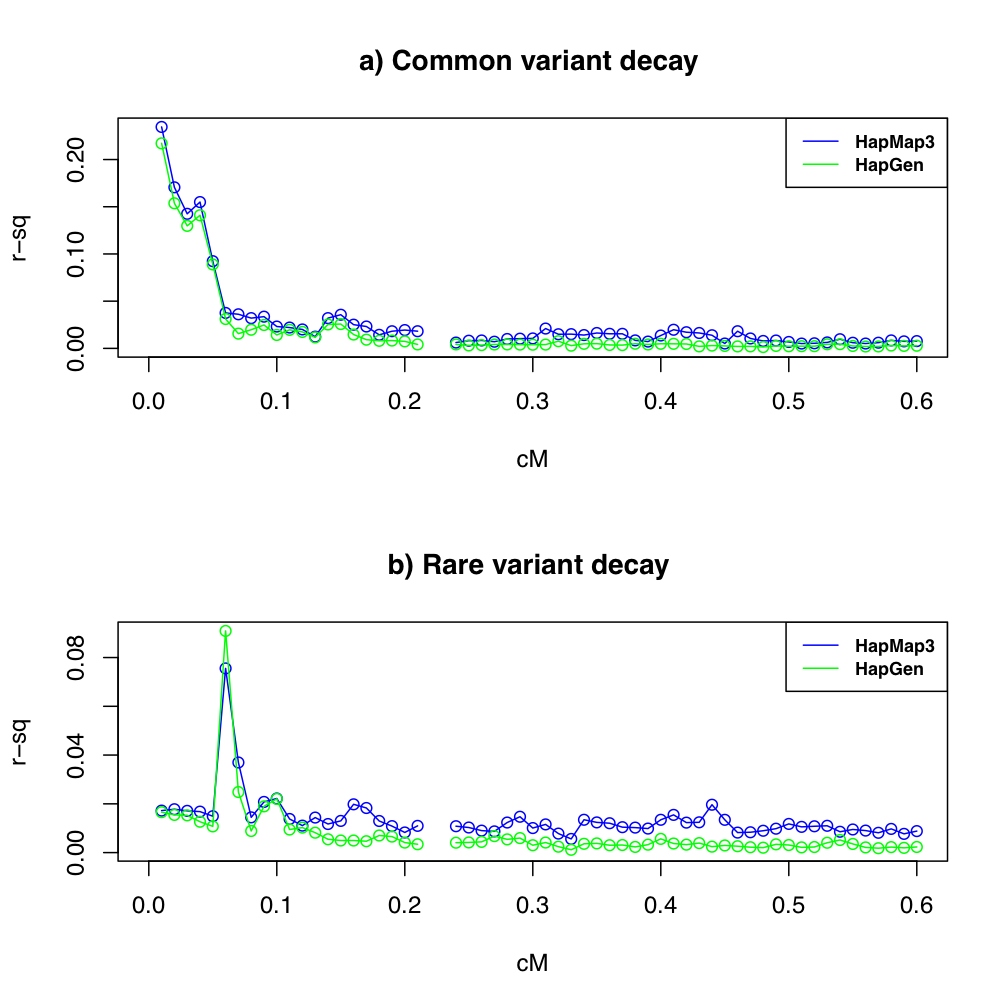

Supplement: Figure S4 — Empirical LD patterns are preserved in HAPGEN simulations. Plotted above is data for region 1 in CEU. For each 0.01 cM bin, the figure presents the mean pair-wise LD (measured in r2) between variants from the resequencing and genotyping data for a) common markers (minor allele frequency >0.04) or b) common and rare markers (minor allele frequency <0.04). We observe that HapMap 3 LD patterns (blue) are largely preserved in HAPGEN simulations (green). Missing points reflect lack of data for certain distance bins. (TIFF) [file pcbi.1002600.s004.tif]

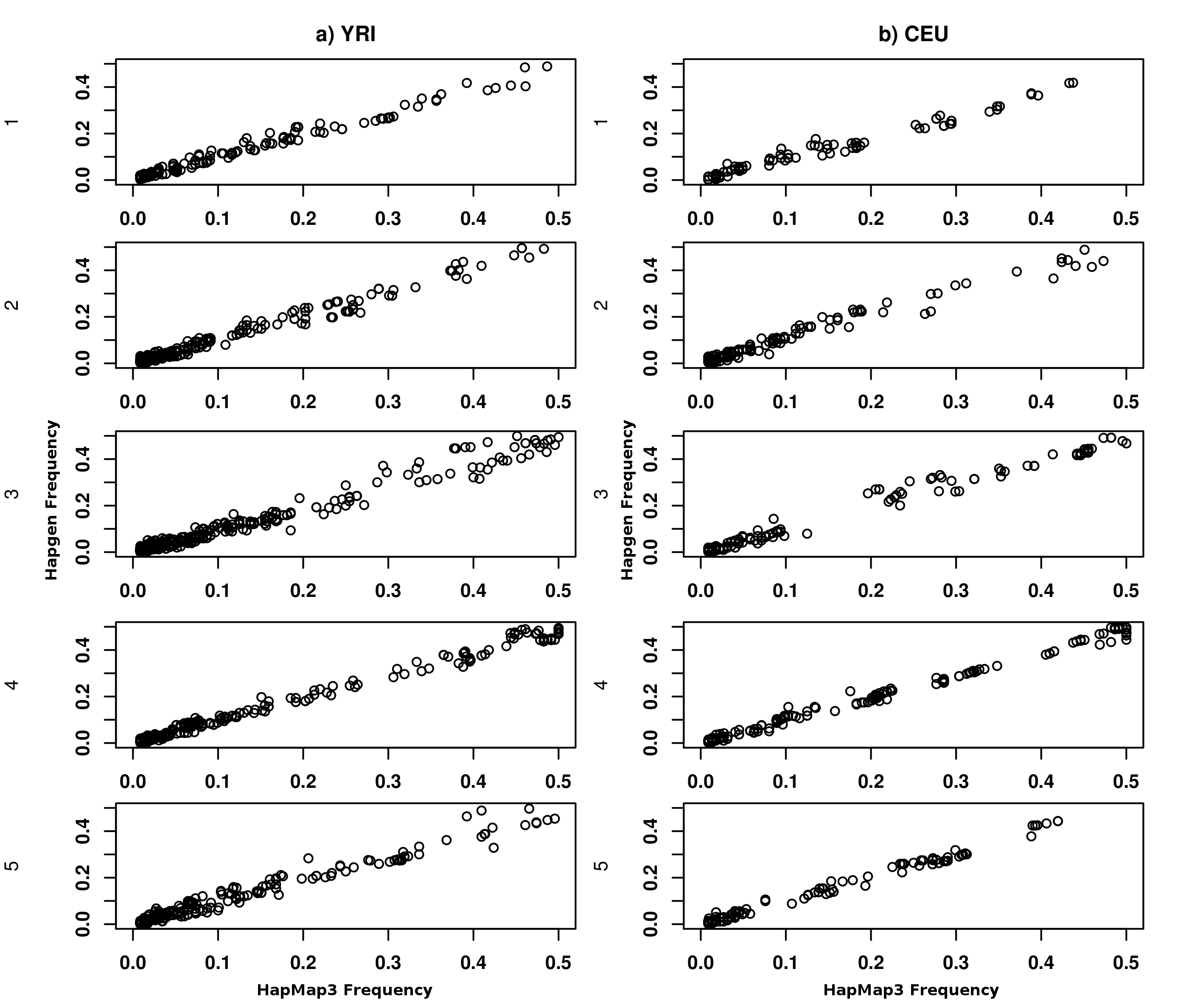

Supplement: Figure S5 — Minor allele frequency in HapMap3 compared to minor allele frequency in HAPGEN simulations. Plotted are minor allele frequencies in HapMap 3 (x-axis) compared to minor allele frequencies in HAPGEN simulations (y-axis) for a) YRI and b) CEU. Each row represents a separate region. No drastic departures from the original minor allele frequencies are observed in the simulated data. (TIFF) [file pcbi.1002600.s005.tif]

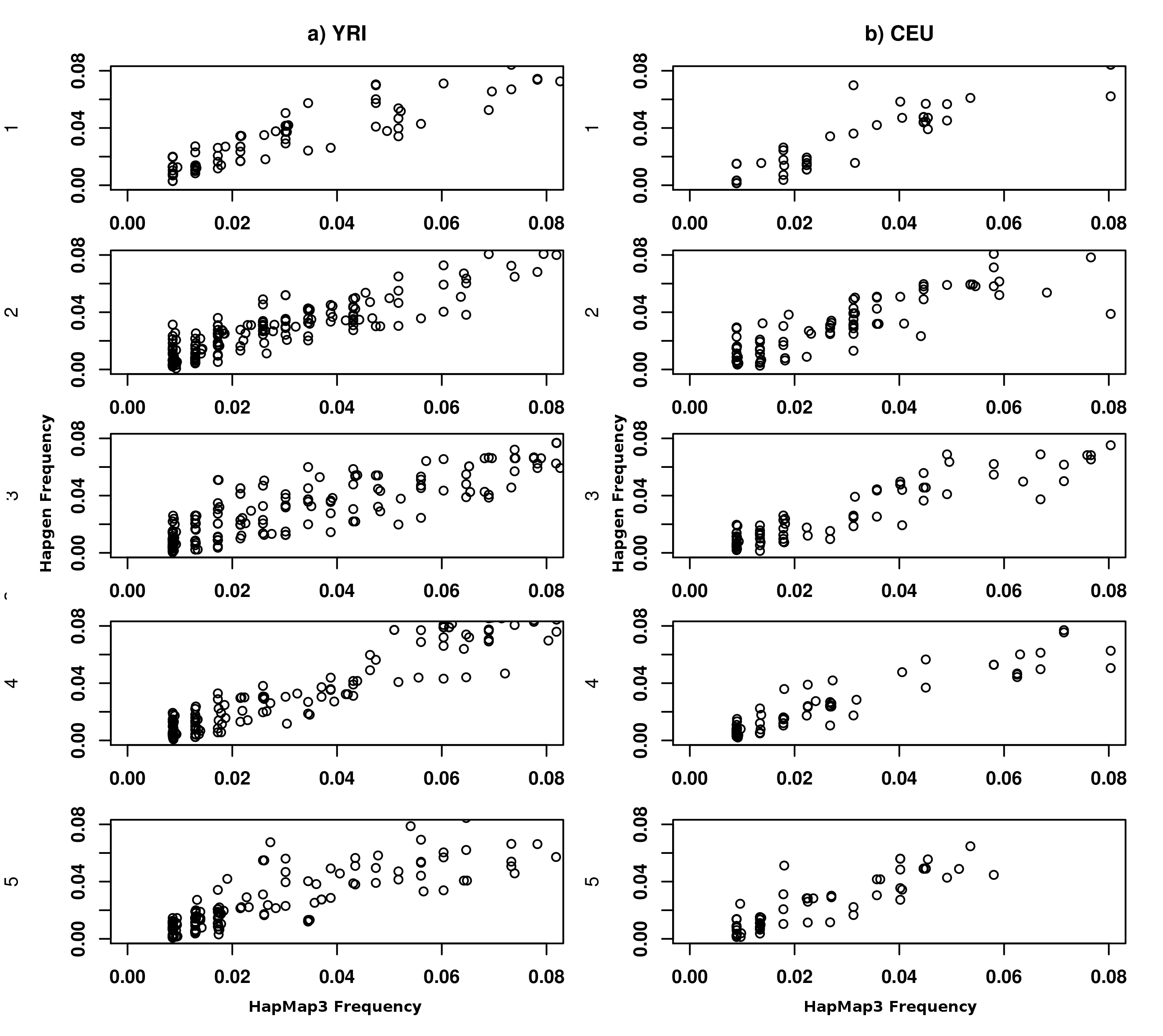

Supplement: Figure S6 — Minor allele frequency in HapMap3 compared to minor allele frequency in HAPGEN simulations for frequencies below 0.08. Same plot as in Figure S5 showing only variants with frequencies below 0.08. As in Figure S5, no drastic departures from the original minor allele frequencies are observed in the simulated data. (TIFF) [file pcbi.1002600.s006.tif]
