# Supplementary material for: Predicting Signatures of “Synthetic Associations” and “Natural Associations” from Empirical Patterns of Human Genetic Variation
Source: PLoS Comput Biol. 2012 Jul 5;8(7):e1002600. doi: 10.1371/journal.pcbi.1002600 (PMC3390358; doi:10.1371/journal.pcbi.1002600)
Supplement: Table S1 — Percentage of tests with significant associations. (DOC) [file pcbi.1002600.s007.doc]

**Table S1**: **Percentage of tests with significant associations.**

|  | **YRI** | | | | |  | **CEU** | | | | |
| --- | --- | --- | --- | --- | --- | --- | --- | --- | --- | --- | --- |
| **Locus #** | **Common (2,1.5)^** | **Common(2,3)^** | **Rare (5)^** | **Rare (9)^** | **Random*** |  | **Common (2,1.5)^** | **Common**  **(2,3)^** | **Rare (5)^** | **Rare (9)^** | **Random*** |
| 1 | 31.40 | 100.00 | 93.80 | 97.20 | 2.00 |  | 67.60 | 100.00 | 91.60 | 95.60 | 2.20 |
| 2 | 19.80 | 99.80 | 98.00 | 97.60 | 2.80 |  | 58.00 | 100.00 | 95.00 | 99.60 | 2.40 |
| 3 | 38.00 | 100.00 | 95.80 | 99.60 | 3.20 |  | 37.60 | 100.00 | 80.00 | 84.60 | 1.20 |
| 4 | 31.80 | 99.80 | 86.40 | 94.40 | 2.00 |  | 54.20 | 100.00 | 89.60 | 96.80 | 2.20 |
| 5 | 28.60 | 100.00 | 94.00 | 97.20 | 1.80 |  | 36.00 | 100.00 | 87.60 | 97.00 | 1.40 |
| **Mean** | **29.92** | **99.92** | **93.60** | **97.20** | **2.36** |  | **50.68** | **100.00** | **88.76** | **94.72** | **1.88** |

^ Corresponds to the notation of Figure 1.

* Corresponds to random phenotypic assignment.
